# Evaluation of Oxidative Stress in Cardiomyocytes during the Aging Process in Rats Treated with Resveratrol

**DOI:** 10.1155/2018/1390483

**Published:** 2018-05-02

**Authors:** P. Aguilar-Alonso, O. Vera-López, E. Brambila-Colombres, O. Segura-Badilla, R. Avalos-López, M. Lazcano-Hernández, A. R. Navarro-Cruz

**Affiliations:** ^1^Departamento de Bioquímica-Alimentos, Facultad de Ciencias Químicas, Edificio 5FCQ-202D, Benemérita Universidad Autónoma de Puebla, Ciudad Universitaria, 72570 Puebla, PUE, Mexico; ^2^Departamento de Análisis Clínicos, Facultad de Ciencias Químicas, Benemérita Universidad Autónoma de Puebla, Puebla, PUE, Mexico; ^3^Facultad de Ciencias de la Salud y de los Alimentos, Departamento de Nutrición y Salud Pública, Programa UBB Saludable, Universidad del Bío-Bío, Concepción, Chile; ^4^Licenciatura en Q.F.B., BUAP, Puebla, PUE, Mexico

## Abstract

The substantial increase in the number of elderly people in our societies represents a challenge for biology and medicine. The societies of the industrialized countries are subject to a progressive aging process that translates into an increase in the cardiovascular risk of the population. In the present work, the activity of catalase and superoxide dismutase was evaluated, as well as markers of oxidative stress (concentration of nitric oxide and total lipoperoxidation in its main components: malondialdehyde and 4-hydroxyalkene) in cardiomyocytes during the aging process in rats treated with resveratrol. Rats were divided into 4 groups according to the following categories: control (without treatment), negative control group (administered with physiological solution with 10% ethanol), positive control group (administered with vitamin E, 2 mg/kg/day), and group administered with resveratrol (10 mg/kg/day); these groups in turn were divided into 2, 4, 6, and 8 months of treatment. The analysis of nitric oxide showed a decreased level in the cardiac tissue in the groups treated with resveratrol; the same occurs when total lipoperoxidation is analyzed. The enzymatic activity studied (catalase and superoxide dismutase) did not present significant changes with respect to the controls. It is concluded that the cardioprotective effect of resveratrol is due to the antioxidant effect and other antiaging effects and not to the activation of the enzymes catalase and superoxide dismutase.

## 1. Introduction

The substantial increase in the number of elderly people in our societies represents a challenge for biology and medicine. The societies of the industrialized countries are subject to a progressive aging process that translates into an increase in the cardiovascular risk of the population. Aging is a multifactorial process in which numerous hypotheses have been postulated in order to explain the degenerative molecular processes that act in it [1, 2]. Harman in 1956 was the first to formulate the hypothesis that the biochemical processes generated largely by the cellular oxidative metabolism are responsible for numerous pathophysiological alterations and that could favor the molecular processes associated with aging [3].

The hypothesis of oxidative stress in aging refers to the completion of the genetic program that governs the sequence and duration of several ontogenetic phases and is linked to the expenditure of a defined sum of energy. The level of oxidative stress depends on the speed of generation of oxidants and antioxidant defense levels, which are genetically controlled but are also influenced by epigenetic factors. This oxidative stress exerts a regulatory influence on gene expression and is different at different stages of development [4]. Numerous studies show that, although the maximum lifetime could be altered by varying the metabolic rate, the total energy expended during life (metabolic potential) remains constant and is a characteristic of the species. A mechanism by which the metabolic rate influences the development and aging can be determined through modulations in the levels of oxidative stress [5].

Oxidative stress is a situation in which the cells are exposed to a prooxidant environment that can affect the homeostasis of the redox state. In parallel, the defensive antioxidant mechanisms were developed to counteract the action of reactive oxygen species (ROS) overgeneration, resulting in a tissue vulnerability against the action of these reactive molecules, which seem to participate in some degenerative processes of biological systems [6].

Resveratrol (3,5,4′-trihydroxy-*trans*-stilbene) is a nonflavonoid natural polyphenol belonging to the family of stilbenes that is produced in 72 plant species in response to an exogenous factor such as UV radiation or pathogens such as bacteria or fungi. It consists of two aromatic rings joined by a methylene bridge [7].

The interest in the cardioprotective nature of resveratrol arose from the study by Douste-Blazy et al. [8], in which cardiovascular risk factors (1985–1987) were studied in two population samples from the French regions of Strasbourg and Toulouse. Resveratrol seems to be, among others, responsible for a nutritional fact called “French paradox”, since the French are Europeans who eat more saturated fats and yet have a lower risk of cardiovascular disease than other Europeans, such as the English, who, like the French, follow a diet rich in saturated fats.  Table 1 summarizes the activities and concentrations at which resveratrol presents cardioprotective activity [9].

From the end of the 80s, a group of biomarkers that directly or indirectly provide information on the concentration of different types of reactive oxygen species (ROS) and nitrogen (RNS) began to be introduced in the measurement of oxidative stress in the human organism [10]. It has even been suggested that there are specific biomarkers for certain diseases [11]. Some of the markers of oxidative stress are listed above.

Nitric oxide (NO^•^): increasing ROS concentrations decrease the amount of bioactive NO^•^ by chemical inactivation to form toxic peroxynitrite (ONOO^−^). Peroxynitrite in turn can “uncouple” the NOS (nitric oxide synthase) endothelial enzyme to become a dysfunctional superoxide-generating enzyme, which contributes to vascular oxidative stress [12].

Malondialdehyde (MDA): it is a final product of oxidation that is generated after the oxidation of biological membranes. This compound is the most common of those known as lipoperoxides and is used as a marker of oxidative stress in plasma and tissues [13–16]. Some works on aging have analyzed the relationship of MDA with age, using MDA and other products of lipoperoxidation as direct markers of oxidative stress [17].

Catalase (CAT): CAT as a part of the antioxidant system is involved in the destruction of H_2_O_2_ generated during cellular metabolism. This enzyme is characterized by its high reaction capacity but relatively little affinity for the substrate [18].

Superoxide dismutase (SOD): these enzymes catalyze the conversion of the superoxide radical (O_2_^•−^) into hydrogen peroxide (H_2_O_2_) and molecular oxygen (O_2_), in one of the fastest catalyzed reactions known [19].

## 2. Materials and Methods

### 2.1. Experimental Animals

Male Wistar rats, 3 months old, were obtained from the Bioterio Claude Bernard of the Benemérita Universidad Autónoma de Puebla. The animals were kept under standard conditions of a bioterium with dark-light cycles of 12 hours and temperature of 21°C, with access to water and food ad libitum. For the experiment, they were divided into 4 groups according to their administration and divided into the following categories: control (without treatment), negative control group (administered with physiological solution with 10% ethanol), positive control group (administered with vitamin E, 2 mg/kg/day), and group administered with resveratrol (10 mg/kg/day), which were distributed to be treated during different periods of time (2, 4, 6, and 8 months). Vitamin E was chosen as a positive control because it is considered the antioxidant par excellence for the human body; the doses used of vitamin E and resveratrol were the ones that reported the best results in the previous study [20].

The administration was carried out in all cases orally (cannula). The resveratrol corresponded to the trademark Lemi & Jo Resveratrol® *(Polygonum cuspidatum)*. All the procedures followed the rules according to the “Guide for the Care and Use of Laboratory Animals” of Mexico and approved by the Institutional Committee for the Care and Use of Animals. All efforts were focused to minimize the suffering of the animals.

### 2.2. Heart Homogenate Obtention

The hearth tissue was weighted and homogenized in PBS solution (pH 7.2–7.4) in a ratio 1 : 4, at 5340 g during 5 minutes in a Tissue-Tearor BioSpec mod. 985370, taking care of cold line in ice; one part of homogenate was separated for SOD activity, then the homogenate was centrifuged at 14850*g* for 30 minutes in a cooled centrifuge, and the supernatant was separated for posterior analysis. The cold chain was maintained (−70°C) as much as possible, and dry ice was used for the thawing on the day the samples were worked. For the homogenate and the supernatant, ice was used to maintain at 4°C on the day of analysis.

### 2.3. Quantification Techniques

#### 2.3.1. Quantification of Nitrites

NO^•^ was determined by nitrite concentration evaluation. The nitrites were measured by using the Griess reaction. Griess reagent was composed of equal volumes of 0.1% N-(1-naphthyl)ethylenediamine dihydrochloride and 1.32% sulfanilamide in 60% acetic acid (Sigma). The colorimetric reaction was made in 100 *μ*L of supernatant and 100 *μ*L Griess reagent. The volume was adjusted to 1 mL by adding distilled water. The absorbance of the samples was determined at 540 nm in a spectrophotometer and compared to a standard curve of NANO_2_ in each assay. Results were expressed as micromoles of nitrite per milligram of protein (*μ*M of NO_2_^−•^/mg of protein) [21].

#### 2.3.2. Quantification of Total Proteins (TP)

It was performed by the Sedmak and Grossberg method [22] using a standard curve of bovine serum albumin as standard. In the case of cardiomyocytes, the proteins were quantified in 1 *μ*L of the supernatant of the homogenate plus 500 *μ*L of the color reagent (Coomassie blue 0.06%), taking it to one mL with distilled water.

#### 2.3.3. Quantification of Malondialdehyde (MDA) plus 4-Hydroxyalkene (4-HDA)

650 *μ*L of N-methyl-2-phenylindole solution was dissolved in a mixture of acetonitrile : methanol (3 : 1), 200 *μ*L of sample and was placed in a vortex for 3-4 seconds. 150 *μ*L of 99% methanesulfonic acid was added and vigorously mixed; the tubes were covered, then incubated at 45°C for 40 minutes. They were cooled to room temperature and centrifuged for 15 minutes at 850*g*. Finally, the absorbance at 586 nm was read against a reagent blank in a spectrophotometer (SpectrumVis SP1105) at 586 nm. The concentration of MDA plus 4-HDA was determined by interpolating the optical density of the samples on a standard curve of 1,1,3,3-tetramethoxypropane (0.5 to 10 *μ*L), which was determined in parallel in each trial.

#### 2.3.4. Quantification of Malondialdehyde (MDA)

650 *μ*L of N-methyl-2-phenylindole solution was dissolved in a mixture of acetonitrile : methanol (3 : 1), 200 *μ*L of sample and was placed in a vibro agitator for 3-4 seconds. 150 *μ*L of 35% of hydrochloric acid was added and vigorously mixed; the tubes were covered, then incubated at 45°C for 60 minutes. They were cooled to room temperature and centrifuged for 15 minutes at 850*g*. Finally, the absorbance at 586 nm was read against a reagent blank in a spectrophotometer (SpectrumVis SP1105) at 586 nm. The concentration of MDA plus 4-HDA was determined by interpolating the optical density of the samples on a standard curve of 1,1,3,3-tetramethoxypropane (0.5 to 10 *μ*L), which was determined in parallel in each trial.

The concentration of 4-hydroxyalkenals was calculated as the difference between the concentrations obtained using methanesulfonic acid minus the concentration obtained using hydrochloric acid [23].

#### 2.3.5. Catalase Activity in Cardiomyocytes

34 *μ*L of supernatant of cardiomyocytes was mixed in a quartz cuvette with 333 *μ*L of hydrogen peroxide; 30 mM plus 50 mM phosphate buffer, pH 7.4, were added. During 2 minutes of reaction, the difference in optical density per minute at 240 nm was determined and compared to a blank without reaction [24].

#### 2.3.6. Superoxide Dismutase (SOD)

100 *μ*L of cardiomyocyte homogenate was mixed in a quartz cuvette with 2.8 mL of tris-HCL buffer solution; 8.20 and 50 *μ*L of EDTA solution were added. 50 *μ*L of the pyrogallol solution was then added, and after 10 seconds of reaction, the optical density difference per minute at 420 nm was determined, against a blank without reaction.

### 2.4. Statistical Analysis

The results are the mean of 7 animals analyzed in triplicate ± the standard deviation (SD). One-way ANOVA and Dunnett's multiple comparison test were used as a posttest to evaluate statistically significant differences with respect to the control group for MDA, MDA plus 4-HDA, 4-HDA, and nitric oxide. An unpaired parametric Student *t*-test analysis of two stems was used for enzymatic activities. Values of *p* < 0.05 were considered significant.

## 3. Results and Discussion

There are still numerous challenges ahead in relation to understanding aging [25, 26]; nevertheless, although the aging process is complex and multifactorial, López-Otín et al. [27] described nine hallmarks that could explain this process: genomic instability, telomere wear, epigenetic alterations, proteostasis loss, dysregulated nutrient detection, dysfunction mitochondrial, cellular senescence, depletion of stem cells, and alteration of intercellular communication. The interesting thing would be that every one of these theories converges at some point on oxidative stress. For example, the mitochondrial theory of free radicals of aging proposes that progressive mitochondrial dysfunction occurs with aging resulting in increased production of ROS, which in turn causes greater mitochondrial deterioration and overall cellular damage [28].

**Figure 1:**
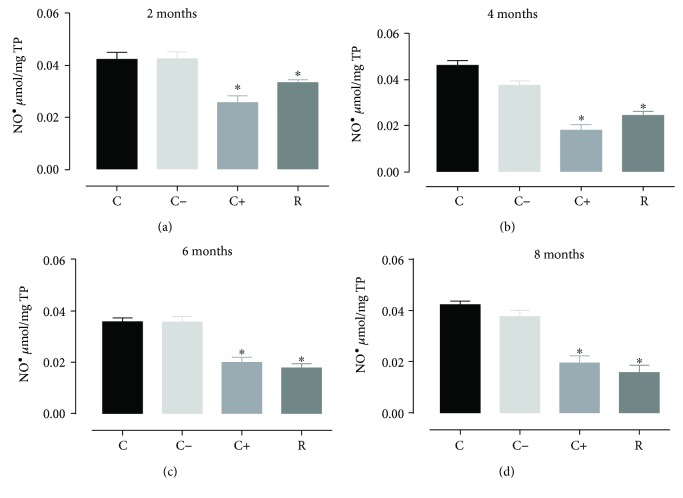
Concentration of nitric oxide in cardiomyocytes during the aging process (2, 4, 6, and 8 months) in rats treated with resveratrol. Results obtained with *n* = 7 animals per group analyzed in triplicate. A one-way ANOVA was realigned, followed by a Dunnett's multiple comparison test, *p* < 0.05. C: control, C−: negative control, C+: positive control, R: resveratrol. ^∗^*p* < 0.05, statistically significant difference with respect to the control group.

Oxidative stress produces changes in the generation of free radicals that accumulate in the aging process; one of the most affected organs is the heart, producing damage in the cardiomyocytes [29], so it was decided to analyze the NO^•^ which normally is a protective vasodilator agent and a second messenger, when is overgenerated in some situations such as aging process, heart and brain ischaemia, hypertension among others is a NRS (nitrogen reactive species) generator of oxidative stress [30]. The results obtained showed a significant decrease (*p* < 0.05) of nitric oxide in all treated groups. The vitamin E group (positive control) tended to decrease nitric oxide levels more, but after 8 months of treatment, both groups (positive control and resveratrol) had the same effect (Figure 1). As observed, the prolonged treatment with resveratrol consecutively decreased nitric oxide levels until 8 months of treatment, when compared with vitamin E (positive control); vitamin E has better antioxidant effect until reaching 8 months of treatment. These results showed that in the longer treatment time (during the aging process), the resveratrol decreases the concentration of NO^•^ in cardiac cells, from 18.49% decrease in the group of 2 months of treatment to 62.67% decrease in the group of 8 months of treatment with resveratrol.

**Figure 2:**
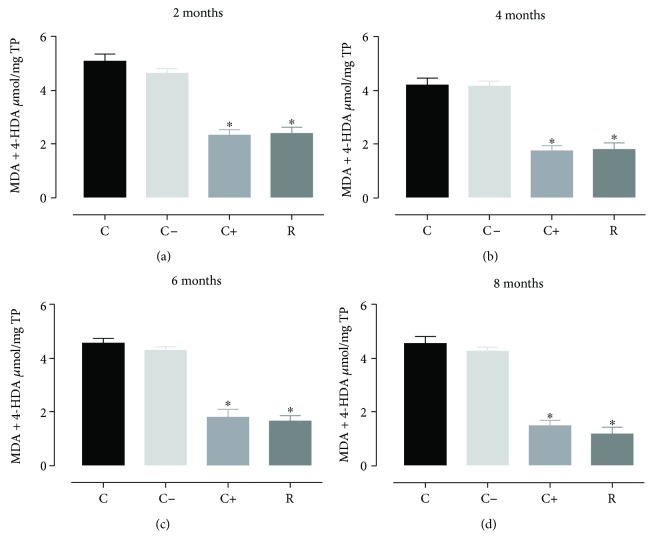
Concentration of MDA plus 4-HDA in cardiomyocytes during the aging process (2, 4, 6, and 8 months) in rats treated with resveratrol. Results obtained with *n* = 7 animals per group analyzed in triplicate. A one-way ANOVA was realigned, followed by a Dunnett's multiple comparison test, *p* < 0.05. C: control, C−: negative control, C+: positive control, R: resveratrol. ^∗^*p* < 0.05, statistically significant difference with respect to the control group.

The decreased concentrations of nitric oxide were also accompanied by the total lipoperoxidation products, which are here represented by its main components malondialdehyde plus 4-hydroxyalquenals during the aging process in rats treated with resveratrol. When reactive species are not scavenged by antioxidant systems, they induce harmful processes to cells such as lipoperoxidation, which involves the conversion of polyunsaturated fatty acids into highly reactive aldehydes [29] which in turn increase oxidative stress.  Figure 2 shows that during the aging process the rats treated with resveratrol presented a significantly decreased production (*p* < 0.05) of the total lipoperoxidation products with respect to the control group and negative control; there were no differences in the resveratrol group with respect to the positive control (vitamin E).

**Figure 3:**
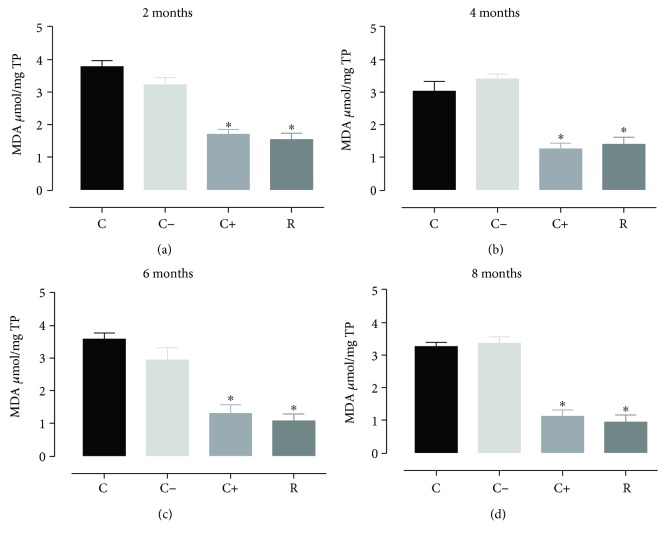
Concentration of MDA in cardiomyocytes during the aging process (2, 4, 6, and 8 months) in rats treated with resveratrol. Results obtained with *n* = 7 animals per group analyzed in triplicate. A one-way ANOVA was realigned, followed by a Dunnett's multiple comparison test, *p* < 0.05. C: control, C−: negative control, C+: positive control, R: resveratrol. ^∗^*p* < 0.05, statistically significant difference with respect to the control group.

**Figure 4:**
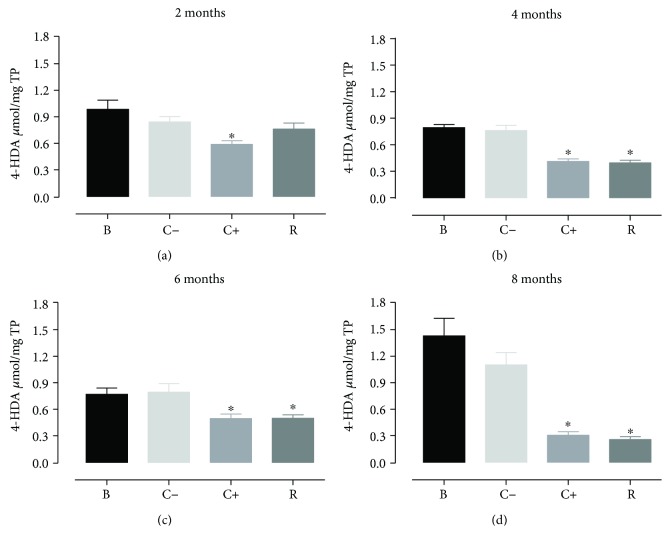
Concentration of 4-HDA in cardiomyocytes during the aging process (2, 4, 6, and 8 months) in rats treated with resveratrol. Results obtained with *n* = 7 animals per group analyzed in triplicate. A one-way ANOVA was realigned, followed by a Dunnett's multiple comparison test, *p* < 0.05. B: white, C−: negative control, C+: positive control, R: resveratrol. ^∗^*p* < 0.05, statistically significant difference with respect to the control group.

With respect to malondialdehyde, it was observed that the aging process in rats treated with resveratrol and the rats treated with vitamin E showed significant decreases in the generation of malondialdehyde (Figure 3) but without differences between these two groups, presenting a similar situation in the levels of 4-hydroxyalkenals (Figure 4).

Regarding the CAT and the SOD, contrasting to results found by Hosoda et al. [31] who reported the decrease of intracellular ROS through the induction of SOD, our findings showed that there were no differences in the levels of SOD or catalase during the study period; however, this must be carefully analyzed, since it is known that when an antioxidant supplementation is used to compensate systemic oxidative stress promoted by an imbalance derived from various oxidants, most of the main antioxidant enzymes are negatively regulated and/or are not activated [32].

No differences were observed neither with respect to the negative control nor with the target (Tables 2 and 3), which shows that the activity of these two enzymes does not change significantly during the aging process and the administration of an exogenous antioxidant (such as resveratrol or vitamin E), but it should also be considered that many studies have been carried out administering mega doses of vitamin E [33] or resveratrol [34]. We worked at resveratrol levels of 10 mg/kg of weight and the amount of vitamin E needed (in this case 2 mg/kg) to cover the recommended daily intakes of vitamin E in rats; the objective was to observe the effect of a chronic dose and not an acute one on oxidative stress.

The comparison of the negative control group (10% ethanol) with the target group did not show significant differences in any of the months of treatment, so it is concluded that the vehicle does not interfere in the normal aging process.

## 4. Conclusion

The administration of resveratrol during the aging process in rat cardiomyocytes showed a significant decrease in the markers of nitric oxide oxidative stress and total lipoperoxidation. The major lipoperoxidation product in heart was malondialdehyde compared to 4-hydroxyalkenals probably due to the presence of the *ω* group fatty acids. The administration of resveratrol during the aging process could help to decrease levels of oxidative stress during the aging process.

Since there are no significant differences in the activity of the enzymes catalase and superoxide dismutase with respect to their targets in cardiomyocytes during the aging process in rats treated with resveratrol, it could be concluded that the antioxidant activity exerted by resveratrol in cardiomyocytes during the process of aging in rats treated with resveratrol is not due to the activation of the enzymes catalase and superoxide dismutase but apparently to the direct antioxidant effect of resveratrol (and vitamin E). However, this result should be analyzed from several perspectives, since the molecular basis of the pharmacological effects of resveratrol is its multiple effects, ranging from a direct physical interaction to indirect modulations such as in the expression levels [35].

## Figures and Tables

**Table 1:** Main cardioprotective activities of resveratrol.

Activity	Concentration	Reference
Reduction of cardiovascular structural and functional deterioration in CHF.	5.0 mg/kg	Ahmet et al. [36]
Attenuation of postinfarct cardiac remodeling and contractile dysfunction.	2.5 mg/kg	Raj et al. [37]
Activation of a novel deacetylating pathway and attenuation of cardiac oxidative stress in diabetic heart.	10.0 mg/kg	Bagul et al. [38]
Induces autophagy and protects hearts from doxorubicin-induced toxicity.	5.0–50.0 mg/kg	Dutta et al. [39]
Prevents oxidative stress induced cardiomyocyte injury mainly by preserving the activities of critical antioxidant enzymes.	2.5 mg/kg	Movahed et al. [40]
Increase of expression of AK1 and IDPm on ventricular modeling.	1.0 mg/kg	Lin et al. [41]
Reverse of abnormalities in diastolic heart function associated with high-fat feeding in obese prone rats.	2.5 mg/kg	Louis et al. [42]
Suppression of sympathetic neural remodeling process after myocardial infarction.	1.0 mg/kg	Xin et al. [43]
Beneficial effects on myocardial function, coronary perfusion, EC function, and vascular tone.	10.0 mg/kg	Joshi et al. [44]
Protection against recurrent stroke.	25.0 mg/kg	Clark et al., [45]

**Table 2:** Catalase activity during aging in rats treated with resveratrol.

	Data					*P* value			
	B	C−	C+	R	C− versus B	C+ versus B	R versus B	C− versus R	R versus C+
2 months									
Mean	83.60	84.13	87.17	92.02	0.882	0.391	0.176	0.148	0.392
Std. deviation	8.47	3.70	6.40	12.98					
4 months									
Mean	51.03	51.6	47.18	44.63	0.908	0.413	0.202	0.596	0.202
Std. deviation	8.59	9.61	8.38	9.14					
6 months									
Mean	62.48	59.98	59.42	53.26	0.465	0.581	0.060	0.209	0.050
Std. deviation	8.27	2.93	11.63	7.48					
8 months									
Mean	33.25	35.76	32.17	34.75	0.396	0.690	0.548	0.600	0.174
Std. deviation	5.37	3.58	3.47	2.56					

B: blank group, C−: negative control group (ethanol 10%), C+: positive control (vitamin E 2 mg/kg), R: resveratrol group (10 mg/kg).

**Table 3:** Superoxide dismutase activity during aging in rats treated with resveratrol.

	Data					*P* value			
	B	C−	C+	R	C− versus B	C+ versus B	R versus B	C− versus R	R versus C+
2 months									
Mean	15.11	14.34	14.24	15.63	0.228	0.128	0.492	0.124	0.094
Std. deviation	1.08	1.12	0.51	1.60					
4 months									
Mean	15.83	15.57	17.21	16.56	0.821	0.184	0.484	0.260	0.313
Std. deviation	2.36	1.84	1.06	1.24					
6 months									
Mean	14.71	15.91	16.04	15.81	0.088	0.142	0.195	0.880	0.780
Std. deviation	1.52	0.78	1.66	1.48					
8 months									
Mean	14.99	15.70	15.75	15.80	0.160	0.120	0.186	0.929	0.931
Std. deviation	0.66	0.90	0.92	1.28					

B: blank group, C−: negative control group (ethanol 10%), C+: positive control (vitamin E 2 mg/kg), R: resveratrol group (10 mg/kg).
